# Sex and age differences in sST2 in cardiovascular disease

**DOI:** 10.3389/fcvm.2022.1073814

**Published:** 2023-01-18

**Authors:** Danielle J. Beetler, Katelyn A. Bruno, Damian N. Di Florio, Erika J. Douglass, Swikriti Shrestha, Carsten Tschöpe, Madeleine W. Cunningham, Jan Krejčí, Julie Bienertová-Vašků, Sabine Pankuweit, Dennis M. McNamara, Eun-Seok Jeon, Sophie van Linthout, Lori A. Blauwet, Leslie T. Cooper, DeLisa Fairweather

**Affiliations:** ^1^Department of Cardiovascular Medicine, Mayo Clinic, Jacksonville, FL, United States; ^2^Center for Clinical and Translational Science, Mayo Clinic, Rochester, MN, United States; ^3^Mayo Clinic Graduate School of Biomedical Sciences, Mayo Clinic, Jacksonville, FL, United States; ^4^Division of Cardiovascular Medicine, Department of Medicine, University of Florida, Gainesville, FL, United States; ^5^Department of Molecular Pharmacology and Experimental Therapeutics, Mayo Clinic, Rochester, MN, United States; ^6^Berlin Institute of Health (BIH) at Charité – Universitätsmedizin Berlin, BIH Center for Regenerative Therapies (BCRT), Berlin, Germany; ^7^German Centre for Cardiovascular Research, Berlin, Germany; ^8^Department of Cardiology, Charité – Universitätsmedizin Berlin, Berlin, Germany; ^9^Department of Microbiology and Immunology, University of Oklahoma Health Sciences Center, Oklahoma City, OK, United States; ^10^First Department of Internal Medicine and Cardioangiology, St. Anne’s University Hospital, Brno, Czechia; ^11^Faculty of Medicine, Masaryk University, Brno, Czechia; ^12^Incubator of Kinanthropology Research, Faculty of Sports, Masaryk University, Brno, Czechia; ^13^Department of Pathological Physiology, Faculty of Medicine, Masaryk University, Brno, Czechia; ^14^Department Internal Medicine-Cardiology, Philipps-University of Marburg, Marburg, Germany; ^15^Division of Cardiology, Department of Medicine, University of Pittsburgh, Pittsburgh, PA, United States; ^16^University of Pittsburgh Medical Center (UPMC), Heart and Vascular Institute, Pittsburgh, PA, United States; ^17^Division of Cardiology, Department of Medicine, Heart Vascular Stroke Institute, Samsung Medical Center, Sungkyunkwan University School of Medicine, Seoul, Republic of Korea; ^18^Olmsted Medical Center, Rochester, MN, United States; ^19^Department of Environmental Health Sciences and Engineering, Johns Hopkins Bloomberg School of Public Health, Baltimore, MD, United States

**Keywords:** biomarkers, heart failure, myocardial infarct, coronary artery disease, cardiomyopathy, congestive heart failure

## Abstract

**Aims:**

The goal of this study was to determine whether sex and age differences exist for soluble ST2 (sST2) for several cardiovascular diseases (CVDs).

**Methods:**

We examined sST2 levels using an ELISA kit for myocarditis (*n* = 303), cardiomyopathy (*n* = 293), coronary artery disease (CAD) (*n* = 239), myocardial infarct (MI) (*n* = 159), and congestive heart failure (CHF) (*n* = 286) and compared them to controls that did not have CVDs (*n* = 234).

**Results:**

Myocarditis occurred in this study in relatively young patients around age 40 while the other CVDs occurred more often in older individuals around age 60. We observed a sex difference in sST2 by age only in myocarditis patients (men aged 38, women 46, *p* = 0.0002), but not for other CVDs. Sera sST2 levels were significantly elevated compared to age-matched controls for all CVDs: myocarditis (*p* ≤ 0.0001), cardiomyopathy (*p* = 0.0009), CAD (*p* = 0.03), MI (*p* = 0.034), and CHF (*p* < 0.0001) driven by elevated sST2 levels in females for all CVDs except myocarditis, which was elevated in both females (*p* = 0.002) and males (*p* ≤ 0.0001). Sex differences in sST2 levels were found for myocarditis and cardiomyopathy but no other CVDs and were higher in males (myocarditis *p* = 0.0035; cardiomyopathy *p* = 0.0047). sST2 levels were higher in women with myocarditis over 50 years of age compared to men (*p* = 0.0004) or women under 50 years of age (*p* = 0.015). In cardiomyopathy and MI patients, men over 50 had significantly higher levels of sST2 than women (*p* = 0.012 and *p* = 0.043, respectively) but sex and age differences were not detected in other CVDs. However, women with cardiomyopathy that experienced early menopause had higher sST2 levels than those who underwent menopause at a natural age range (*p* = 0.02).

**Conclusion:**

We found that sex and age differences in sera sST2 exist for myocarditis, cardiomyopathy, and MI, but were not observed in other CVDs including CAD and CHF. These initial findings in patients with self-reported CVDs indicate that more research is needed into sex and age differences in sST2 levels in individual CVDs.

## 1. Introduction

Myocarditis is an important cause of sudden death and chronic heart failure with an estimated worldwide prevalence of 0.5–4.0% (1–3). We showed previously that the heart failure biomarker soluble ST2 (sST2) (which is also called interleukin-1 receptor-like 1/IL1RL1) was significantly elevated in the sera of patients with clinically suspected and biopsy confirmed myocarditis, but only in men with myocarditis that were under 50 years of age (4). Sera sST2 levels were found to correlate with New York Heart Association (NYHA) classification in young men with myocarditis, but not left ventricular (LV) ejection fraction (LVEF) obtained from echocardiography (4). These findings highlight the importance of examining biomarkers according to sex and age. Note that sST2/IL1RL1, which is located on human chromosome 2 and is a member of the IL-1R/Toll-like receptor (TLR)4 family (sST2, GeneID: 9173), is consistently confused with the protein suppression of tumorigenicity 2 (especially in the cardiovascular literature) which is located on human chromosome 11 (ST2, GeneID: 6761) and is associated with cancer.

The ST2 gene (IL1RL1 GeneID: 9173) encodes 4 isoforms of ST2 by alternative splicing: ST2L, a transmembrane receptor for interleukin (IL)-33; sST2, a secreted soluble form of ST2; ST2V, a variant of ST2 found in the gut; and ST2LV, which is secreted by the eye, heart, lung, and liver (5–7), sST2 mainly acts as a decoy receptor to dampen IL-33 cytokine activity by binding to the cytokine (8) but some recent findings indicate that it might also function as an extracellular ligand by binding to an unknown receptor, suggesting a secondary function as an inflammatory mediator or growth factor instead of only as a decoy receptor (9, 10). sST2 is a cytokine that has been shown to be induced by biomechanical strain in cardiac fibroblasts, cardiomyocytes, and vascular endothelial cells that binds IL-33, thereby inhibiting its effect (11, 12). In translational studies we found that serum sST2 levels are associated with increased myocardial inflammation and are increased by testosterone in male mice with viral myocarditis but unaffected by estradiol in females (4). Serum IL-33 and sST2 have been associated with more severe autoimmune diseases (i.e., Sjögren’s disease, myocarditis), asthma, and cancers, but use of knockout mice and recombinant protein or blocking antibody in animal models have yielded conflicting results (13–15). Almost no animal studies have taken into consideration sex, age, or mouse strain in relation to the effect of these cytokines. Although serum sST2 has been clearly demonstrated to display sex differences during myocarditis and chronic heart failure (HF), there is little data available that examines sex and age differences in sST2 levels for specific CVDs, especially for acute or sub-chronic stages of CVD.

Two recent studies examined sera levels of sST2 in large cohorts of patients to determine whether sex-specific cutoffs would enhance determination of risk for hospitalization or death (16, 17). The study by Harmon et al. examined participants from the Rochester Epidemiology Project for cardiovascular outcomes. They found sex-specific differences in sST2 associated with incident HF, HF with reduced EF (HFrEF), HF with preserved EF (HFpEF), major adverse cardiac events (MACE), and mortality (16). Vergaro et al. examined 13 cohorts of patients with stable chronic HF that were part of the Biomarkers In Heart Failure Outpatient Study (BIOS) consortium. Patients were classified as having HFrEF (LVEF ≤ 40%), HF with mildly reduced LVEF (HFmrEF, LVEF 41–49%) or HFpEF (LVEF ≥ 50%) according to the European Society of Cardiology (ESC) guidelines (18, 19). In 4,540 patients with chronic HF, they found that HFrEF was more prevalent in men (88% vs. 73%, *p* < 0.001), while HFmrEF and HFpEF occurred more often in women (10% vs. 7% and 17% vs. 5%; both *p* < 0.001), and men had significantly higher serum levels of sST2 and high sensitivity cardiac troponin T than women but there were no sex differences in NT-proBNP (17). Another large study by Suthahar et al. examined sex differences in primary CVD biomarkers including sST2 from several large cohorts and reported that sST2 was elevated in the sera of men with chronic HF and that there was an interaction between sex and age (13). Although previous studies examined age or sex, none of the studies specifically examined the effect of age on sST2 levels according to sex. The purpose of this study was to examine whether sex and age differences exist in serum sST2 levels in several acute CVDs such as myocarditis, cardiomyopathy, atherosclerosis/coronary artery disease (CAD), and myocardial infarct (MI) as well as congestive HF (CHF). Here we would like to gain insight by examining the effect of sex and age on sST2 levels by comparing a number of CVDs.

## 2. Materials and methods

### 2.1. Ethics statement

Research carried out in this study complied with the Helsinki Declaration. This study was approved by the Mayo Clinic Institutional Review Board (IRB). Receipt of a waiver of the need to consent subjects was obtained for Mayo Clinic Biobank samples (IRB No: 08-007049, 16-010321). Approval was obtained from the local IRB or ethics board at each institution for studies involving patients including Johns Hopkins Bloomberg School of Public Health (IRB No: 00003212), Mayo Clinic (IRB No: 08-002108, 12-003533, 14-008222, 17-004148), University of Oklahoma Health Sciences Center (IRB No: 2712, 7449), St. Anne’s University Hospital Brno (Reference No: 19V/2014), and Charite University of Medicine in Berlin (EA2/140/16). Patients from Marburg were included within Subproject 9 of the German Competence Network Heart Failure, which conformed to the principles outlined in the Declaration of Helsinki and was approved by the German Heart Failure Network and the local ethics committee. Informed written consent or IRB approved waiver of consent was obtained from all patients.

### 2.2. Patients and controls

Patients with myocarditis (*n* = 303) were identified from existing registries/biobanks in the United States, Germany, Korea, and the Czechia, as previously (4). Patients were included in the study based on a diagnosis of clinically suspected myocarditis according to the 2013 European Society of Cardiology position statement and heart failure symptoms of <6 months duration. Sera were collected during the patient’s first visit and analyzed by enzyme-linked immunoabsorbent assay (ELISA). Cardiac function was assessed for all patients with myocarditis using echocardiography and/or cardiac magnetic resonance imaging. Heart failure symptoms were categorized by NYHA classification. Testosterone, 17β-estradiol, medication history, and menopause status were not available for any of the patients.

Patients with cardiomyopathy (*n* = 293), CAD (*n* = 239), MI (*n* = 159), or CHF (*n* = 286) were obtained through the Mayo Clinic Biobank based on ICD-9/10 codes for individual CVDs. Biobank patients self-reported their cardiovascular condition. Biobank sera samples were stored at −80°C and sST2 analyzed by enzyme-linked immunoabsorbent assay (ELISA) at Mayo Clinic Florida. Testosterone, 17β-estradiol, medication history, and menopause status were not available for any of the patients.

Controls (*n* = 234) were obtained through the Mayo Clinic Biobank and had no ICD-9/10 codes for CVDs; however, we could not rule out other chronic inflammatory conditions. Control samples without any known condition were not available. Upon analysis it was noted that some control patients displayed sex differences, and some had sST2 values that were much higher than expected for no CVD status. In a large control reference population (*n* = 1136) from the Framingham Heart Study, male sex (*p* < 0.0001) and older age (*p* = 0.004) predicted higher serum sST2 levels (20). As the Mayo Clinic Biobank is based on self-reported disease status, it could be that some of the control patients had unreported or unknown underlying CVD, an infection, or another inflammatory condition. sST2 levels are known to increase during infection and during many inflammatory conditions such as asthma and autoimmune diseases (14). Compared to reference/control sST2 levels established for a similarly aged, larger USA population [8.6–49.3 and 7.2–33.5 ng/mL for males and females, respectively (21)], our controls of both sexes displayed ranges with higher maximum sST2 levels. Statistical outlier exclusion did not alleviate the disparities between our controls and the average sST2 reference levels reported by Lu et al. in 2010, but when we removed values above 49.3 ng/mL for males and 33.5 ng/mL for females, our average sST2 values for controls became 30.2 ng/mL for males and 22.8 ng/mL for females. Although these values are still higher than the average sST2 reference values developed by Lu et al. (male: 24.9 ng/mL, female: 16.9 ng/mL) (21), they were under the Critical Diagnostics/Presage human sST2 ELISA kit cutoff for heart failure of >35 ng/mL (22, 23).

Patient demographics are described in Tables 1–4. For biobank controls, cardiac function values (LVEF and NYHA class) were obtained from the Mayo Clinic EMR for individual patients by biobank staff and provided for analysis de-identified. For patients with myocarditis (*n* = 303), cardiac function was assessed at each site (US, Germany, Korea, Czechia) using echocardiography and/or cardiac magnetic resonance imaging and HF symptoms categorized by NYHA class and provided for analysis de-identified. Sera samples were stored at −80°C locally and sST2 analyzed by ELISA at each site. Age 50 years was chosen for analyzing the effect of age in women because we did not have information on menopause status and thus age is often used as a surrogate when menopause status is not known (24–26). We examined data according to age 50 in men because we analyzed data in that manner for women. Additionally, age < 50 years has been used in the literature to define young patients in the context of CVD (27, 28).

**TABLE 1:** Demographics of controls and patients with CVDs according to sex (*n* = 1346, 60.8% male).

	Total	CVD type	Combined	Men (M)		Women (W)		M vs. W
		*n*	Mean ± SD^a^	*n*	Mean ± SD	*n*	Mean ± SD	*P*-value^b^
Age	*n* = 1,346 Mean 61.4 ± 16.8 Range 18–91	Con (*n* = 234)	64.7 @ 11.8	116	65.8 @ 12.7	118	63.6 @ 10.9	0.29
		Myo (*n* = 303)	40.1 @ 14.6	237	38.4 @ 13.6	66	46.4 @ 16.3	**0.0002**
		Card (*n* = 293)	67.1 @ 11.4	182	68 @ 11.6	111	65.4 @ 10.9	0.26
		CAD (*n* = 239)	67.2 @ 12.5	115	67.1 @ 12.8	124	67.4 @ 12.3	0.99
		MI (*n* = 159)	68.5 @ 10.5	121	69.3 @ 9.6	38	65.9 @ 12.7	0.35
		CHF (*n* = 286)	70 @ 10.7	166	70.9 @ 9.8	120	68.8 @ 11.7	0.35
BMI	*n* = 1,023 Mean 29.2 ± 6	Con (*n* = 226)	27.4 @ 4.7	113	28.1 @ 4.5	113	26.7 @ 4.9	0.052
		Myo (n/a)	−	−	−	−	−	−
		Card (*n* = 290)	29.3 @ 6.2	179	29.7 @ 5.4	111	28.8 @ 7.3	0.73
		CAD (*n* = 233)	29.3 @ 6.2	112	29.9 @ 4.9	121	28.8 @ 6.8	0.58
		MI (*n* = 156)	29.7 @ 6	118	29.5 @ 4.9	38	30.2 @ 8.6	0.983
		CHF (*n* = 281)	30.6 @ 6.5	161	30.7 @ 5.7	120	30.4 @ 7.4	0.99
LVEF%	*n* = 1,086 Mean 54.7 ± 15.1	Con (n/a)	−	−	−	−	−	−
		Myo (*n* = 283)	48.4 @ 20.3	220	48.4 @ 20.3	60	48.3 @ 20.6	0.99
		Card (*n* = 293)	53 @ 14.1	182	51.1 @ 13.9	111	56.2 @ 13.9	**0.013**
		CAD (*n* = 237)	62.1 @ 7.4	115	61.4 @ 8.4	122	62.8 @ 6.4	0.55
		MI (*n* = 158)	53.1 @ 11.6	120	52.3 @ 11.9	38	55.6 @ 10.4	0.49
		CHF (*n* = 286)	52.4 @ 13.8	166	48.8 @ 13.6	120	57.4 @ 12.4	**<0.000001**

^a^BMI, body mass index; CAD, coronary artery disease; Card, cardiomyopathy; CHF, congestive heart failure; Con, control; CVD, cardiovascular disease; M, men; MI, myocardial infarction; Myo, myocarditis; n/a, not available; LVEF, left ventricle ejection fraction; SD, standard deviation; W, women. ^b^*P*-values with significance < 0.05 indicated in bold font.

**TABLE 2:** Race demographics of controls and patients with CVDs according to sex (*n* = 1043).

	Total (*n* = 1,043)	White	Black/African American	Asian	Mixed	Unknown or missing
		**(*n*)%**	**(*n*)%**	**(*n*)%**	**(*n*)%**	**(*n*)%**
Total	Con^a^ (*n* = 234)	(234) 100%	(0) 0%	(0) 0%	(0) 0%	(0) 0%
	Myo (n/a)	–	–	–	–	–
	Card (*n* = 293)	(266) 90.8%	(4) 1.4%	(1) 0.3%	(19) 6.5%	(3) 1.0%
	CAD (*n* = 239)	(238) 99.6%	(0) 0%	(0) 0%	(1) 0.4%	(0) 0%
	MI (*n* = 159)	(147) 92.5%	(2) 1.3%	(1) 0.6%	(7) 4.4%	(2) 1.3%
	CHF (*n* = 286)	(286) 100%	(0) 0%	(0) 0%	(0) 0%	(0) 0%
Men	Con (*n* = 116)	(116) 100%	(0) 0%	(0) 0%	(0) 0%	(0) 0%
	Myo (n/a)	–	–	–	–	–
	Card (*n* = 182)	(169) 92.9%	(2) 1.1%	(0) 0%	(9) 4.9%	(2) 1.1%
	CAD (*n* = 115)	(114) 99.1%	(0) 0%	(0) 0%	(1) 0.9%	(0) 0%
	MI (*n* = 121)	(110) 90.9%	(2) 1.7%	(1) 0.8%	(7) 5.8%	(1) 0.8%
	CHF (*n* = 146)	(146) 100%	(0) 0%	(0) 0%	(0) 0%	(0) 0%
Women	Con (*n* = 118)	(118) 100%	(0) 0%	(0) 0%	(0) 0%	(0) 0%
	Myo (n/a)	–	–	–	–	–
	Card (*n* = 111)	(97) 87.4%	(2) 1.8%	(1) 0.9%	(10) 9.0%	(1) 0.9%
	CAD (*n* = 124)	(124) 100%	(0) 0%	(0) 0%	(0) 0%	(0) 0%
	MI (*n* = 38)	(37) 97.4%	(0) 0%	(0) 0%	(0) 0%	(1) 2.6%
	CHF (*n* = 120)	(120) 100%	(0) 0%	(0) 0%	(0) 0%	(0) 0%

^a^CAD, coronary artery disease; Card, cardiomyopathy; CHF, congestive heart failure; Con, control; CVD, cardiovascular disease; MI, myocardial infarction; Myo, myocarditis; n/a, not available.

**TABLE 3:** NYHA class in patients with CVDs (*n* = 678).

	Total (*n* = 678)	Class I (*n* = 200)	Class II (*n* = 196)	Class III (*n* = 236)	Class IV (*n* = 46)
		**(*n*)%**	**(*n*)%**	**(*n*)%**	**(*n*)%**
Individual NYHA^a^ class	Myo (*n* = 267)	(42) 15.7%	(133) 49.6%	(77) 28.7%	(15) 5.6%
	Card (*n* = 147)	(69) 46.9%	(18) 12.2%	(50) 34.0%	(10) 6.8%
	CAD (*n* = 33)	(14) 42.4%	(6) 18.2%	(13) 39.4%	(0)
	MI (*n* = 61)	(25) 41.0%	(7) 11.5%	(22) 36.1%	(7) 11.5%
	CHF (*n* = 170)	(50) 29.4%	(32) 18.8%	(74) 43.5%	(14) 8.2%
		**NYHA Class I–II (*n* = 396)**		**NYHA Class III–IV (*n* = 282)**	
		**(*n*)%**		**(*n*)%**	
Combined NYHA class	Myo (*n* = 267)	(175) 65.3%		(92) 34.3%	
	Card (*n* = 147)	(87) 59.2%		(60) 40.8%	
	CAD (*n* = 33)	(20) 60.6%		(13) 39.4%	
	MI (*n* = 61)	(32) 52.5%		(29) 47.5%	
	CHF (*n* = 170)	(82) 48.2%		(88) 51.8%	

^a^CAD, coronary artery disease; Card, cardiomyopathy; CHF, congestive heart failure; Con, control; CVD, cardiovascular disease; MI, myocardial infarction; Myo, myocarditis; NYHA, New York Heart Association.

**TABLE 4:** Heart failure status of patients with CVDs according to LVEF (*n* = 1086).

	Total (*n* = 1,086)	Healthy, LVEF^a^ ≤ 45% (*n* = 342)		HF, LVEF > 45% (*n* = 912)
		**(*n*)%**		**(*n*)%**
LVEF ≤ 45 vs. > 45	Myo (*n* = 280)	(122) 43.6%		(158) 56.4%
	Card (*n* = 293)	(84) 28.7%		(209) 71.3%
	CAD (*n* = 237)	(7) 3.0%		(230) 97.0%
	MI (*n* = 158)	(43) 27.2%		(115) 72.8%
	CHF (*n* = 286)	(86) 30.1%		(200) 69.9%
	**Total (*n* = 1086)**	**HFrEF, LVEF ≤ 40% (*n* = 251)**	**HFmrEF, LVEF 41–49% (*n* = 138)**	**HFpEF, LVEF ≥ 50% (*n* = 869)**
		**(*n*)%**	**(*n*)%**	**(*n*)%**
LVEF by ESC guidelines	Myo (*n* = 280)	(107) 38.2%	(21) 7.5%	(152) 54.3%
	Card (*n* = 293)	(54) 18.4%	(46) 15.7%	(193) 65.9%
	CAD (*n* = 237)	(4) 1.7%	(6) 2.5%	(227) 95.8%
	MI (*n* = 158)	(26) 16.5%	(23) 14.6%	(109) 69.0%
	CHF (*n* = 286)	(60) 21.0%	(38) 13.3%	(188) 65.7%

^a^CAD, coronary artery disease; Card, cardiomyopathy; CHF, congestive heart failure; Con, control; CVD, cardiovascular disease; MI, myocardial infarction; Myo, myocarditis; n/a, not applicable; LVEF, left ventricle ejection fraction.

### 2.3. ELISAs

Enzyme-linked immunoabsorbent assays (ELISAs) provide key quantitative analyses of specific protein levels in biological samples. In this study, patient sera samples were tested in one of two types of sST2 ELISA kits. For all control and CVD samples except myocarditis, the US Food and Drug Administration-approved Critical Diagnostics/Presage human sST2 ELISA kit (REF#BC-1065E, San Diego, CA, USA) was analyzed to determine sST2 levels in the samples according to manufacturer instructions. As this kit was not available when myocarditis samples were initially analyzed, the human R&D Systems sST2 ELISA kit (Cat# DST200, Minneapolis, MN, USA) was used for myocarditis samples. For a subset of myocarditis samples (*n* = 21) the human Critical Diagnostics Presage sST2 kit was compared with the R&D Systems kit to develop a conversion between the kits, as previously (4). The average sST2 value for the R&D Systems kit was 19.45 ng/mL while the Critical Diagnostics kit was 48.39 ng/mL for the same samples. This is a factor of 2.488. The conversion did not change the distribution of the data (Supplementary Figure 1). This conversion was used to calculate sST2 levels for the myocarditis samples to compare levels to other CVDs using the same scale as the Presage kit.

### 2.4. Statistical analysis

Clinical data are expressed as violin plots, so that the distribution of sST2 values within a group can be visualized. With violin plots the median is displayed as the thick dotted line and quartiles Q1 and Q3 are displayed as thin dotted lines. Two-group analysis of normally distributed data were performed using an unpaired Student’s *t*-test. Welch’s test was used for data with unequal variance comparing two groups. Multiple comparisons were analyzed by 1- or 2-way ANOVA. *Post hoc* pairwise comparisons were calculated using Sidak’s or Tukey’s multiple comparisons test. Associations were analyzed using Pearson’s correlation. Categorical correlation data were analyzed using simple linear regression analysis. A value of *p* < 0.05 was considered significant.

## 3. Results

Demographic data of controls and CVD patients are shown in Tables 1, 2. The age of control subjects that did not have CVDs was matched closely to CVD patients (average age in 60s) (Table 1). In contrast, patients with myocarditis had an average age in the 40s in this study. Myocarditis is a CVD known to occur predominantly in young adults (4). A sex difference in age in our study was observed only in men with myocarditis (average age 38) who were significantly younger than women (average age 46) (*p* = 0.0002), but not for other CVDs (Table 1). There were no significant differences between men and women in body mass index (BMI) in controls or CVD groups (Table 1). Men with cardiomyopathy (*p* = 0.013) or CHF (*p* < 0.000001) had worse cardiac function based on LVEF than women (Table 1). Race was mainly white for controls and CVDs (Table 2). Studies from Mayo Clinic have been reported to have low ethnic diversity (29). However, all groups had a similar ethnic diversity for this study. Patient numbers according to New York Heart Association (NYHA) class are shown in Table 3 and patients separated according to LVEF categories in Table 4.

### 3.1. sST2 increased in cardiovascular diseases

**FIGURE 1:**
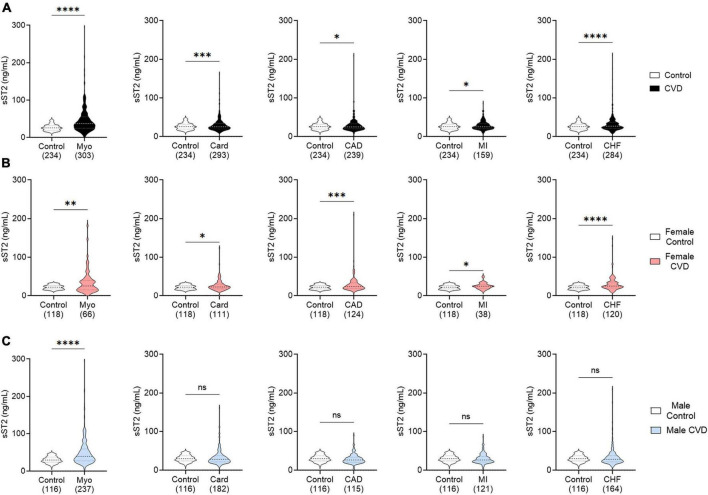
Serum sST2 increased in CVDs. Serum sST2 levels in **(A)** men and women (black), **(B)** women only (peach), or **(C)** men only (blue) were compared between control patients without known CVDs (white) to those with myocarditis (Myo), cardiomyopathy (Card), coronary artery disease (CAD), myocardial infarct (MI), or congestive heart failure (CHF). Violin plots denote data distribution for each group with *p*-value calculated using Welch’s *t*-test: *, *p* < 0.05; ^**^, *p* < 0.01; ^***^, *p* < 0.001; ^****^, *p* < 0.0001; ns, not significant.

Compared to controls, sera sST2 was found to be elevated in all the CVDs that we examined including myocarditis (*p* ≤ 0.0001), cardiomyopathy (*p* = 0.0009), CAD (*p* = 0.03), MI (*p* = 0.034), and CHF (*p* < 0.0001) (Figure 1A). When we compared female controls to females with individual CVDs we found that sera sST2 levels were significantly elevated during disease for myocarditis (*p* = 0.002), cardiomyopathy (*p* = 0.014), CAD (*p* = 0.0009), MI (*p* = 0.028), and CHF (*p* < 0.0001) (Figure 1B). In contrast, we found that sera sST2 levels were only elevated in men with myocarditis compared to controls (*p* ≤ 0.0001), but not for other CVDs (Figure 1C).

### 3.2. Sex differences in sST2 in CVDs

**FIGURE 2:**
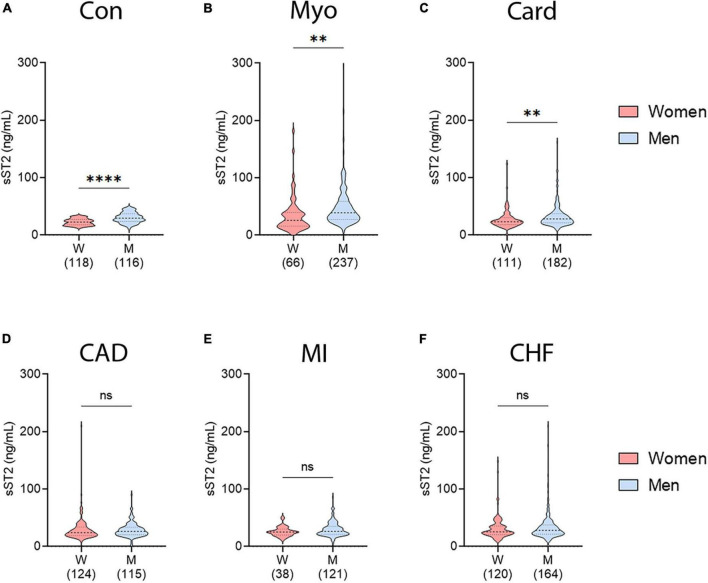
Sex differences in sST2 in controls and CVDs. sST2 levels compared women (peach) vs. men (blue) for **(A)** controls, **(B)** myocarditis (Myo), **(C)** cardiomyopathy (Card), **(D)** coronary artery disease (CAD), **(E)** myocardial infarction (MI), or **(F)** congestive heart failure (CHF). Violin plots denote data distribution for each group with *p*-value calculated using unpaired Student’s *t*-test: ^**^, *p* < 0.01; ^****^, *p* < 0.0001; ns, not significant.

When we examined whether sex differences in sera sST2 levels occurred in our study, we found that sST2 levels were higher in men than women for controls (*p* ≤ 0.0001) as well as for patients with myocarditis (*p* = 0.0035) and cardiomyopathy (*p* = 0.0047) (Figures 2A–C), which includes dilated cardiomyopathy which is a sequelae of myocarditis in susceptible individuals (30–32). However, we did not observe sex differences in sST2 levels in patients with CAD (*p* = 0.77), MI (*p* = 0.087), or CHF (*p* = 0.30) (Figures 2D–F).

### 3.3. Sex and age differences in sST2

We showed previously that sera sST2 levels were higher in men with myocarditis that were under 50 years of age (4). Many conditions where inflammation plays a central role such as autoimmune and cardiovascular diseases, are affected by age as well as sex (4, 33). A recent large-scale analysis of sST2 found that there was a sex-age interaction with sex significantly altering the effect of age (13). Age 50 was chosen as a surrogate timepoint for the menopause transition because it is often used when specific information on menopause status of the patient is unknown (24–26). Males were treated in the same manner as females, and it is known that both estrogens and testosterone decrease with age (27, 28).

**FIGURE 3:**
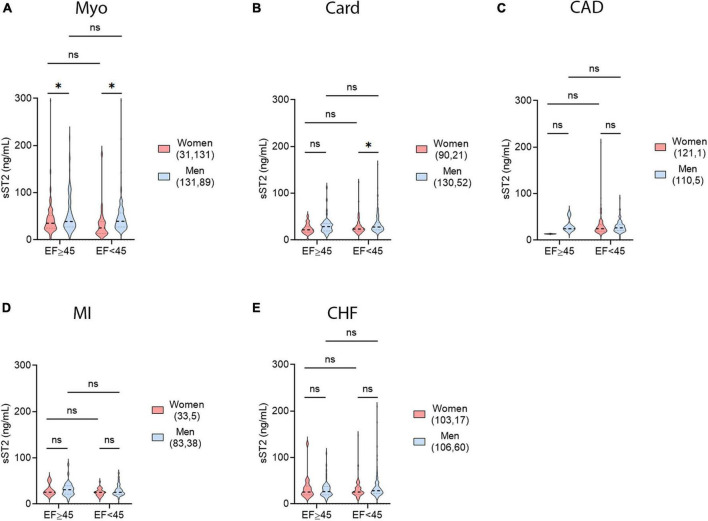
Sex and age differences in sST2 in CVDs. sST2 levels compared women (peach) vs. men (blue) that were <50 vs. ≥50 years of age for **(A)** myocarditis (Myo), **(B)** cardiomyopathy (Card), **(C)** coronary artery disease (CAD), **(D)** myocardial infarction (MI), or **(E)** congestive heart failure (CHF). Violin plots denote data distribution for each group. One-way ANOVA: **(A)** *p* = 0.0043, **(B)** *p* = 0.035, **(C)** *p* = 0.94, **(D)** *p* = 0.16, and **(E)** *p* = 0.70 with two-way comparisons analyzed using unpaired Student’s *t*-test: *, *p* < 0.05; ^***^, *p* < 0.001; ns, not significant. **(A)** Two-way ANOVA *p*-values indicate no interaction between sex and age in patients with myo (*p* = 0.11), but interactions between sex and sST2 (*p* = 0.0047) and age and sST2 (*p* = 0.0496). **(B)** Two-way ANOVA *p*-values indicate no interactions between sex and age (*p* = 0.43) or age and sST2 (*p* = 0.99) in patients with card, but interaction between sex and sST2 (*p* = 0.04). **(C)** Two-way ANOVA *p*-values indicate no interactions between sex and age (*p* = 0.60), age and sST2 (*p* = 0.79), or sex and sST2 (*p* = 0.79) in patients with CAD. **(D)** Two-way ANOVA *p*-values indicate no interactions between sex and age (*p* = 0.32), age and sST2 (*p* = 0.25), or sex and sST2 (*p* = 0.92) in patients with MI. **(E)** Two-way ANOVA *p*-values indicate no interactions between sex and age (*p* = 0.87), age and sST2 (*p* = 0.54), or sex and sST2 (*p* = 0.57) in patients with CHF.

We found that serum sST2 levels were significantly higher in men with myocarditis that were under 50 years of age compared to women under 50 (*p* = 0.0004) (Figure 3A). In contrast, women with myocarditis that are over 50 years of age were found to have significantly elevated sST2 levels compared to younger women (<50 years old) (*p* = 0.015) (Figure 3A). There were no significant differences in sST2 levels in men according to sex and age. These findings parallel the demographics of cohorts with myocarditis, where young women are most protected from myocarditis and young men are at highest risk. Additionally, older women lose cardioprotection against myocarditis and thus their risk of disease increases after age 50 (4). In cardiomyopathy patients, older men had significantly higher levels of sST2 than women (*p* = 0.012) (Figure 3B). Cardiomyopathy occurs more often in men and is more prevalent and severe in older patients (31, 34). Similar to cardiomyopathy, males with MI that were over 50 years of age had significantly higher sST2 levels than females (*p* = 0.043) (Figure 3D). There were no sex and age differences in CAD or CHF (Figures 3C, E); however, the number of patients that were in the <50 years of age group for all diseases except myocarditis was too low to draw conclusions. These diseases are known to occur predominantly in older individuals compared to myocarditis which occurs primarily in young adults. The number of patients in >50 years of age groups were sufficient to draw conclusions about sex differences, and there were no sex differences in CAD and CHF in patients > 50 years of age (Figures 3C, E).

### 3.4. sST2 levels in pre- vs. post-menopausal women with CVDs

**FIGURE 4:**
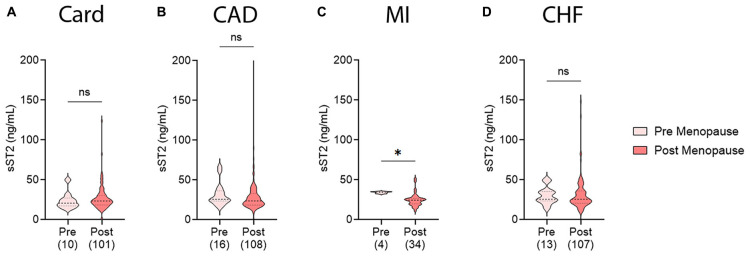
Soluble ST2 (sST2) in pre- vs. post-menopausal women with CVDs. sST2 levels compared pre-menopausal women (light peach) to post-menopausal women (dark peach) for **(A)** cardiomyopathy (Card), **(B)** coronary artery disease (CAD), **(C)** myocardial infarction (MI), or **(D)** congestive heart failure (CHF). Violin plots denote data distribution for each group with *p*-value calculated using unpaired Student’s *t*-test: *, *p* < 0.05; ns, not significant.

To further examine sex differences in sera sST2 levels, we investigated whether menopause status effected biomarker levels in female patients with our CVDs of interest. We separated patients into pre- vs. post-menopausal groups based on self-reported menopause status. Menopause status was not available for the myocarditis patients. No significant differences in sST2 levels were observed in any CVD in this study between pre- vs. post-menopausal women (Figures 4A–D). However, these conditions are known to occur in older patients and the number of patients in the pre-menopause group was likely too low to draw conclusions.

### 3.5. sST2 levels in women with early menopause

**FIGURE 5:**
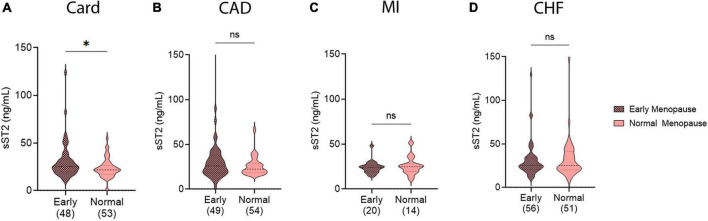
Soluble ST2 (sST2) levels in women with early menopause. sST2 levels compared women with early menopause (Early, magenta) vs. those with normal menopause (peach) for **(A)** cardiomyopathy (Card), **(B)** coronary artery disease (CAD), **(C)** myocardial infarction (MI), or **(D)** congestive heart failure (CHF). Violin plots denote data distribution for each group with *p*-value calculated using unpaired Student’s *t*-test: *, *p* < 0.05; ns, not significant.

Some women experience early menopause through complete cessation of ovulation or removal of ovaries and/or uterus. To test if this early menopause, and thus early loss of natural estrogen cycling, affect sera sST2 levels in a number of CVDs, post-menopausal women were separated into early vs. normal menopause groups based on age or cause of menopause. This information was not available for patients with myocarditis. In women with cardiomyopathy, sST2 levels were higher for post-menopausal women who experienced early menopause versus those who experienced menopause at a natural age range (*p* = 0.02) (Figure 5A). No other CVD showed significant differences in sST2 levels based on an early vs. normal menopause transition (Figures 5B–D).

### 3.6. Sex differences in ejection fraction and sST2 levels

**FIGURE 6:**
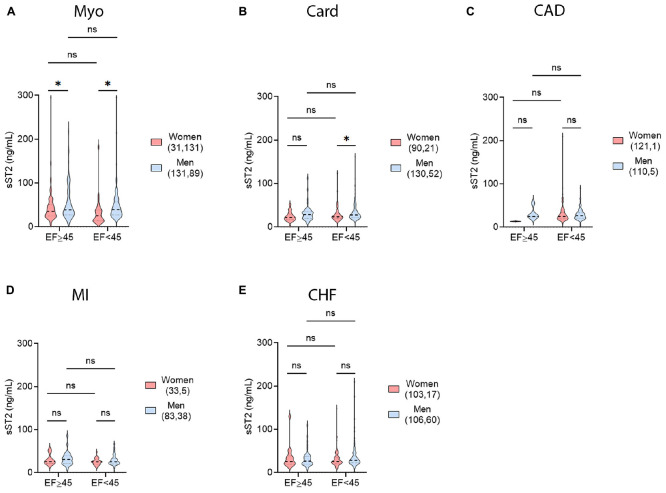
Sex differences in LVEF and sST2. sST2 levels compared women (peach) vs. men (blue) with a left ventricular ejection fraction (LVEF) of ≥45% vs. <45% for **(A)** myocarditis (Myo), **(B)** cardiomyopathy (Card), **(C)** coronary artery disease (CAD), **(D)** myocardial infarction (MI), or **(E)** congestive heart failure (CHF). Violin plots denote data distribution for each group. One-way ANOVA: **(A)** *p* = 0.024, **(B)** *p* = 0.023, **(C)** *p* = 0.79, **(D)** *p* = 0.17, and **(E)** *p* = 0.36 with two-way comparisons analyzed using unpaired Student’s *t*-test: *, *p* < 0.05; ns, not significant. **(A)** Two-way ANOVA *p*-values indicate no interaction between EF and sex (*p* = 0.59) in patients with myocarditis, but interactions between LVEF and sST2 (*p* = 0.04) and sex and sST2 (*p* = 0.0036). **(B)** Two-way ANOVA *p*-values indicate no interactions between EF and sex (*p* = 0.81) or EF and sST2 (*p* = 0.22) in patients with cardiomyopathy, but interactions between sex and sST2 (*p* = 0.01). **(C)** Two-way ANOVA *p*-values indicate no interactions between EF and sex (*p* = 0.34), EF and sST2 (*p* = 0.48), or sex and sST2 (*p* = 0.40) in patients with CAD. **(D)** Two-way ANOVA *p*-values indicate no interactions between EF and sex (*p* = 0.77), EF and sST2 (*p* = 0.23), or sex and sST2 (*p* = 0.39) in patients with MI. **(E)** Two-way ANOVA *p*-values indicate no interactions between EF and sex (*p* = 0.21), EF and sST2 (*p* = 0.87), or sex and sST2 (*p* = 0.85) in patients with CHF.

**FIGURE 7:**
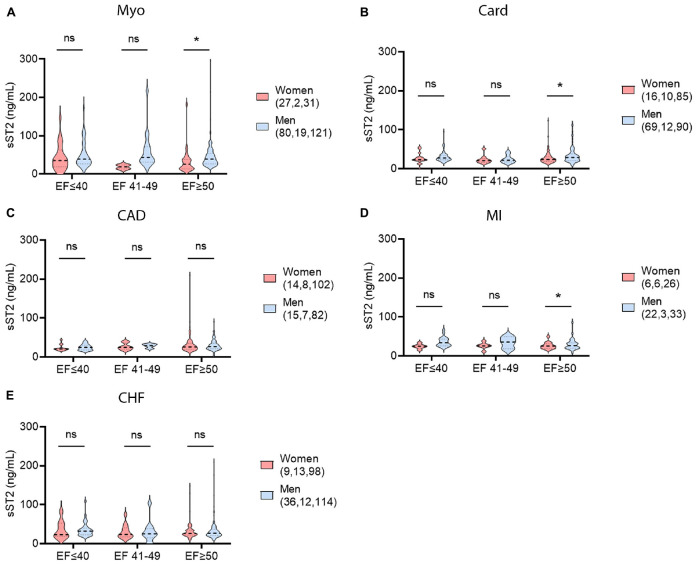
Sex differences in LVEF and sST2 using ESC guidelines. sST2 levels compared women (peach) vs. men (blue) in patients with HFrEF, HFmrEF, or HFpEF for **(A)** myocarditis (Myo), **(B)** cardiomyopathy (Card), **(C)** coronary artery disease (CAD), **(D)** myocardial infarction (MI), or **(E)** congestive heart failure (CHF). Violin plots denote data distribution for each group. One-way ANOVA: **(A)** *p* = 0.053, **(B)** *p* = 0.032, **(C)** *p* = 0.72, **(D)** *p* = 0.093, and **(E)** *p* = 0.89 with two-way comparisons analyzed using unpaired Student’s *t*-test: *, *p* < 0.05; ns, not significant. **(A)** Two-way ANOVA *p*-values indicate no interaction between sex and ejection fraction (EF) in patients with myocarditis (*p* = 0.51), or EF and sST2 (*p* = 0.29), but interaction between and sex and sST2 (*p* = 0.025). **(B)** Two-way ANOVA *p*-values indicate no interactions between sex and EF (*p* = 0.69), EF and sST2 (*p* = 0.16), or sex and sST2 (*p* = 0.15) in patients with cardiomopathy. **(C)** Two-way ANOVA *p*-values indicate no interactions between sex and EF (*p* = 0.89), EF and sST2 (*p* = 0.20) or sex and sST2 (*p* = 0.81) in patients with CAD. **(D)** Two-way ANOVA *p*-values indicate no interactions between sex and EF (*p* = 0.42) and EF and sST2 (*p* = 0.77), but interaction between sex and sST2 (*p* = 0.032) in patients with MI. **(E)** Two-way ANOVA *p*-values indicate no interactions between sex and EF (*p* = 0.95), EF and sST2 (*p* = 0.68), or sex and sST2 (*p* = 0.84) in patients with CHF.

Left ventricle ejection fraction (LVEF) is the most frequently used measure to determine HF for CVDs in many centers. We examined data using a cutoff of LVEF < 45% in order to compare to our previous findings with myocarditis (4). In CVD patients with a LVEF > 45%, only patients with myocarditis displayed sex differences in sST2 levels with women having higher levels (*p* = 0.04) (Figure 6A). In HF patients with LVEF < 45%, men with myocarditis or cardiomyopathy had significantly higher sST2 levels (*p* = 0.032 or *p* = 0.016, respectively) (Figures 6A, B). There were no other significant differences in sST2 and LVEF by sex for any of the other CVDs (Figures 6C–E). We also examined whether sex differences existed for patients according to the recent ESC guidelines: HFrEF if they had a LVEF ≤ 40%, HFmrEF if they had a LVEF of 41–49% or HFpEF if they had a LVEF ≥ 50% (18, 19). There were significant differences in sST2 between men and women with a LVEF > 50% for patients with myocarditis (*p* = 0.047), cardiomyopathy (*p* = 0.012), or MI (*p* = 0.039) (Figure 7).

### 3.7. sST2 relationship to ejection fraction by sex

**FIGURE 8:**
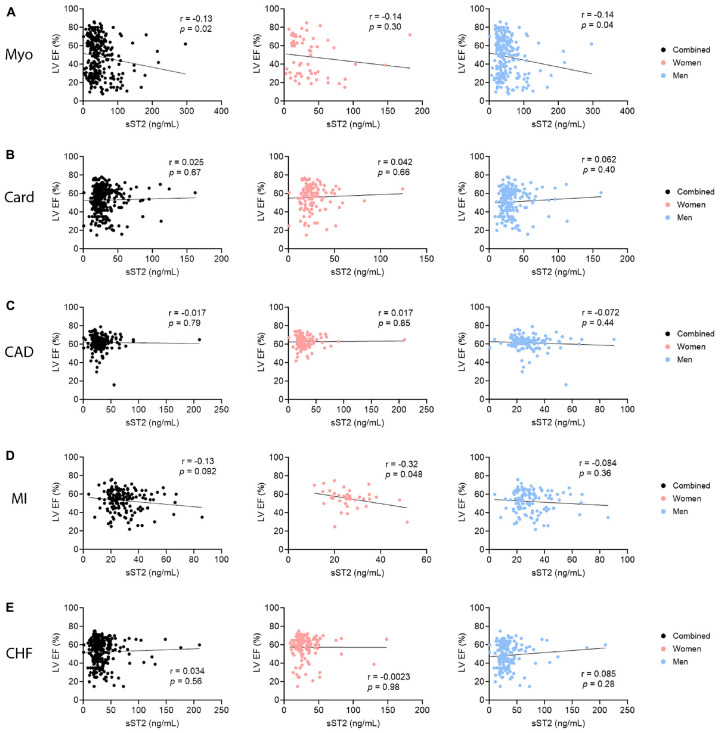
Soluble ST2 (sST2) relationship to LVEF by sex. sST2 levels in men and women (black), women only (peach), or men only (blue) were correlated to % left ventricular ejection fraction (LVEF) for **(A)** myocarditis (Myo), **(B)** cardiomyopathy (Card), **(C)** coronary artery disease (CAD), **(D)** myocardial infarction (MI), or **(E)** congestive heart failure (CHF). *P*-values were calculated using 2-tailed Pearson correlation.

To test if sST2 levels were related to LVEF in any of the CVDs, linear correlations of percent LVEF and sST2 level were conducted in men and women combined and separately by sex for each CVD. In myocarditis patients, LVEF and sST2 displayed a negative correlation, with higher sST2 levels correlating with lower LVEF (*p* = 0.02) (Figure 8A). This trend was driven by male patients (*p* = 0.04), as female patients showed no correlation in sST2 level and LVEF (Figure 8A). In cardiomyopathy and CAD patients, there was no correlation between LVEF and sST2 level in men and women combined or according to sex (Figures 8B, C). MI patients did show a negative correlation between sST2 level and LVEF in women (*p* = 0.048); however, this was masked when men and women were examined together by the lack of correlation between sST2 and LVEF in men (Figure 8D). CHF patients did not show a correlation in sST2 levels and LVEF (Figure 8E).

### 3.8. Sex differences in NYHA class and sST2 levels

**FIGURE 9:**
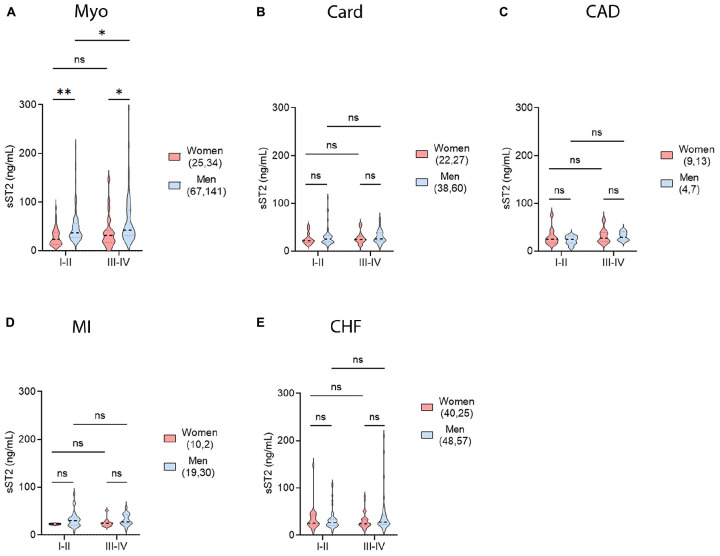
Sex differences in NYHA class and sST2 levels. sST2 levels compared women and men (gray), women (peach), or men (blue) to New York Heart Association (NYHA) class I–II vs. III–IV heart failure for **(A)** myocarditis (Myo), **(B)** cardiomyopathy (Card), **(C)** coronary artery disease (CAD), **(D)** myocardial infarction (MI), or **(E)** congestive heart failure (CHF). Violin plots denote data distribution for each group. One-way ANOVA: **(A)** *p* = 0.0002, **(B)** *p* = 0.66, **(C)** *p* = 0.64, **(D)** *p* = 0.55, and **(E)** *p* = 0.28. **(A)** Two-way ANOVA *p*-values indicate no interaction between NYHA Class and sex (*p* = 0.59) in patients with myocarditis, but interactions between NYHA Class and sST2 (*p* = 0.04) and sex and sST2 (*p* = 0.0002). **(B)** Two-way ANOVA *p*-values indicate no interactions between NYHA Class and sex (*p* = 0.94), NYHA Class and sST2 (*p* = 0.74), or sex and sST2 (*p* = 0.22) in patients with cardiomyopathy. **(C)** Two-way ANOVA *p*-values indicate no interactions between NYHA Class and sex (*p* = 0.63), NYHA Class and sST2 (*p* = 0.32), or sex and sST2 (*p* = 0.52) in patients with CAD. **(D)** Two-way ANOVA *p*-values indicate no interactions between NYHA Class and sex (*p* = 0.75), NYHA Class and sST2 (*p* = 0.80), or sex and sST2 (*p* = 0.20) in patients with MI. **(E)** Two-way ANOVA *p*-values indicate no interactions between NYHA Class and sex (*p* = 0.12), NYHA Class and sST2 (*p* = 0.72), or sex and sST2 (*p* = 0.40) in patients with CHF. *, *p* < 0.05; **, *p* < 0.01.

New York Heart Association (NYHA) class provides another measure of heart function in CVD patients. Higher NYHA class is associated with worse overall cardiac function and physical performance. To increase the statistical power the lower NYHA classes (I–II) were grouped and their sST2 levels compared to those of the higher NYHA classes (III–IV) for each CVD by sex. In myocarditis patients, sST2 values were significantly higher in men with either NYHA class I–II or III–IV HF compared to women (*p* = 0.0026, *p* = 0.033, respectively) (Figure 9A). Men with more severe HF based on NYHA class had higher sST2 levels than men with less severe disease (*p* = 0.012) (Figure 9A). None of the other CVDs studied showed a significant sex difference in HF based on NYHA class (Figures 9B–E).

### 3.9. sST2 relationship to NYHA class by sex

**FIGURE 10:**
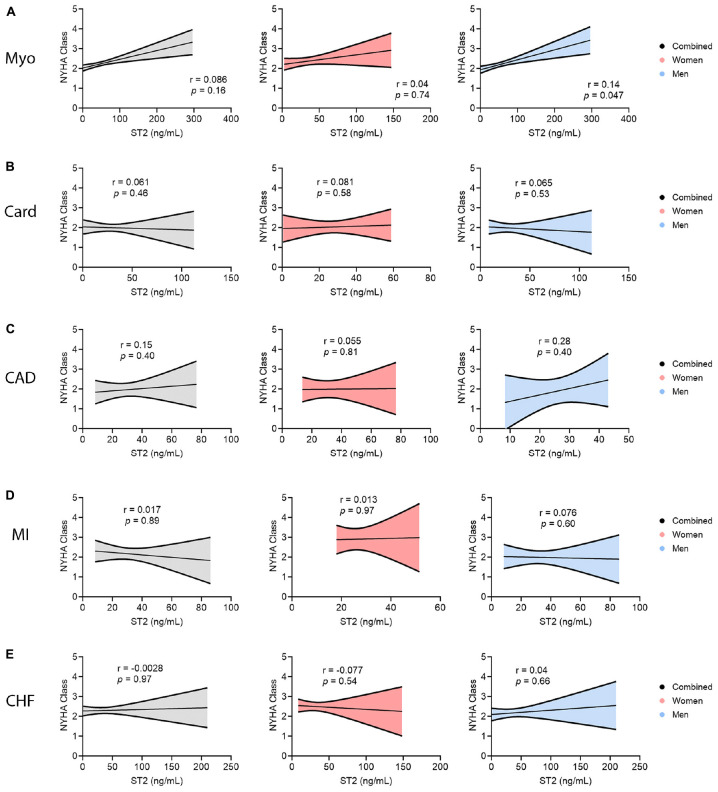
Soluble ST2 (sST2) relationship to NYHA class by sex. sST2 levels compared women (peach) vs. men (blue) with New York Heart Association (NYHA) class I–II vs. III–IV heart failure for **(A)** myocarditis (Myo), **(B)** cardiomyopathy (Card), **(C)** coronary artery disease (CAD), **(D)** myocardial infarction (MI), or **(E)** congestive heart failure (CHF). Data show simple linear regression of data for each group with 95% confidence intervals showing spread of certainty in the model. *P*-values were calculated using 2-tailed Pearson correlation.

To test whether sST2 levels were related to NYHA class in individual CVDs, linear correlations of NYHA class and sST2 level were conducted for men and women combined and separately for each sex and each CVD (Figure 10). The 95% confidence intervals for these correlations were also included to indicate at which relative sST2 values the correlations were most predictive of HF. Out of all the linear correlations conducted, only men with myocarditis displayed a positive correlation for more severe HF indicated by NYHA class, with sST2 level increasing as NYHA class increased (*p* = 0.047) (Figure 10A). sST2 is most predictive for patients with myocarditis around an sST2 level of 80 ng/mL. We did not find a correlation between sST2 level and NYHA class for cardiomyopathy, CAD, or MI, but the linear regressions looked most predictive at sST2 levels < 50 ng/mL (Figures 10B–D). CHF also did not reveal a correlation between NYHA class and sST2 level, but linear regressions looked most predictive between sST2 levels of 50–75 ng/mL (Figure 10E).

## 4. Discussion

Although there have been significant advances in the diagnosis and treatment of HF, the condition remains associated with high rates of hospitalization and mortality worldwide (35–37). Sera sST2 has been found to be a powerful predictor of mortality where a change in sera sST2 levels over time predicts NYHA class III or IV HF, subsequent mortality, and the need for transplantation (38–41). Elevated sera sST2 values have been shown to significantly correlate with both LVEF and NYHA class, although only weakly with LVEF (4, 39, 42). Many clinical studies have confirmed that sera sST2 is a powerful predictor of mortality independently and when combined with N-terminal pro-brain natriuretic peptide (NT-proBNP) in patients with HF and LV systolic dysfunction with a LVEF ≤ 45% (38–41). Because many past studies used a LVEF ≤ 45% as a cutoff and to correspond to our previous publication on myocarditis (4), we examined the relationship of sST2 to heart failure using LVEF ≤ 45%. We also analyzed sST2 levels according to sex using the refined ESC definition of HF (18, 19).

Echocardiography is an important tool used to measure LVEF and HF for most hospitals and centers (43, 44). A recent assessment of nearly 5,000 patients with HF found that men had lower LVEF values that were associated with more hospitalization for heart failure and HF-related mortality (45). In this study we show that elevated serum sST2 levels correlate with lower LVEF and higher NYHA class and HF in patients with clinically suspected or biopsy confirmed myocarditis, but only in males (Figures 8A, 10A). Traditional echocardiography measurements are known to be an insensitive measure of cardiac function and future studies should examine the relationship of serum sST2 to echocardiography-derived global longitudinal strain (GLS) (46), which is likely to be a better measure of early and mild cardiac dysfunction (i.e., HFpEF, LVEF ≥ 50%). We published previously that elevated serum sST2 levels correlate with worse disease in myocarditis patients with NYHA class III-IV HF, but only in males (4). These data indicate the importance of analyzing sST2 and other HF biomarkers according to sex. Recent studies have demonstrated that sST2 and other HF biomarkers like high sensitivity cardiac troponin T (hs-cTnT) differ by sex and that sex-specific cutoffs are needed to enhance determination of risk for hospitalization or death from HF (16, 17).

Most studies examining sST2 and HF have not reported data according to sex and age. In this study we found sex and age differences in serum sST2 levels for myocarditis, cardiomyopathy, and MI. Males with myocarditis and cardiomyopathy had higher levels of sST2 and worse HF. However, after age 50 sST2 levels increased in females with myocarditis, while males over 50 years of age with cardiomyopathy or MI had higher levels of sST2. These data indicate that there are important differences that occur during aging in men and women related to sST2. Changes in sex hormone levels or ratios are one important contributor to sex differences in sST2. We showed previously that testosterone increases serum sST2 levels in male mice with coxsackievirus-induced myocarditis, but estrogen (17β-estradiol) did not alter sST2 levels (4). A study that examined the sera of healthy patients found a similar result that sera estrogen levels did not correlate to sera sST2 levels in females while sST2 levels were affected by testosterone in males, similar to our findings in the mouse model (47). Evidence that estrogen/s may be protective comes from our finding that women with cardiomyopathy and early menopause had higher sST2 levels (Figure 5) indicating that estrogen may reduce sST2 levels. A recent study found that women with early or surgical menopause had higher Framingham Risk Scores for CVD than those with normal menopause (48). But other factors may contribute to age-related changes in sST2 in men and women such as shifts over time in mitochondrial function, which has been associated with HF (49).

A number of studies have found that individuals without known CVDs have elevated levels of sST2 and that levels are higher in men than women (4, 13, 20, 47, 50, 51). In a large reference population (*n* = 1136) from the Framingham Heart Study, sera sST2 levels were found to be significantly elevated in healthy men compared with healthy women where male sex (*p* < 0.0001) and older age (*p* = 0.004) predicted higher serum sST2 levels (20). In another study of 528 healthy men and women, sST2 was found to be significantly elevated in men compared with women (47). In this study we also found that sST2 levels from apparently healthy individuals from the Mayo Clinic Biobank also had elevated sST2 levels and that males had higher levels than females (*p* < 0.0001).

Soluble ST2 (sST2) levels have been found to consistently be elevated in disease states such as infections, CVDs, asthma and autoimmune diseases, and where studies have been conducted, to display sex differences (4, 13, 14). Sex differences in sST2 may be explained at least in part by the role of ST2 in inflammatory responses. IL-33 and ST2/sST2 are members of the IL-1 receptor family that include Toll-like receptor (TLR)4. IL-33 binds the ST2 receptor (ST2L) found on many cell types including innate immune cells like mast cells and macrophages that can drive T helper (Th)1, Th2, and Th17-type immune responses, although the pathway most associated with IL-33/ST2 signaling are Th2 responses and remodeling (52–57). In the context of CVD, Th1- and Th17-associated cytokines increase acute inflammation while Th2- and Th17-associated cytokines promote remodeling and fibrosis, all of which are known to influence the pathogenesis of disease in myocarditis leading to DCM as well as CAD/MI (13, 14, 58–62). IL-33 signaling *via* the ST2 receptor increases inflammation in many auto/inflammatory diseases such as rheumatoid arthritis, inflammatory bowel disease, Sjögren’s disease, and asthma (52–54). In animal models, sST2 has been found to be released predominantly from CD11b+ and CD4+ immune cells (52), which are the primary cardiac immune cells observed during acute myocarditis in mice and humans (32, 58, 63). IL-33/ST2 signaling has been found to upregulate CD11b, TLR4, caspase-1, and IL-1β in a number of human and animal studies of infection and autoimmune disease (52–54). Previously we reported that CD11b, TLR4, caspase-1, and IL-1β were increased by testosterone in male mice with myocarditis (32, 58, 63). We previously showed that treatment of male mice with recombinant IL-33 during initiation of myocarditis significantly increased perimyocarditis resulting in eosinophilic myocarditis (55). We also showed that serum sST2 levels are increased by IL-1β and that there is a direct association between cardiac IL-1β levels and sST2 levels in the heart during myocarditis in male BALB/c mice (4). TLR4 which leads to IL-1β production is a critical pathway in the pathogenesis of myocarditis, CAD, and MI (59, 61–63), and it may be this interaction between TLR4/IL-1β signaling on CD11b + immune cells (i.e., mast cells, macrophages) that elevates sST2 levels in males, where the ST2 receptor is cleaved to form sST2 in an effort to counteract IL-33. Evidence for this hypothesis comes from previous studies that showed that circulating IL-1β levels correlate with NYHA classification and predict mortality in heart failure patients (20). Inflammation was determined to be the best predictor of death or heart transplantation following acute myocarditis based on endomyocardial biopsies (30). Bartunek et al. found that ST2 production did not correlate with wall stress but rather that endothelial cells and/or inflammation contributed to the release of sST2 (64). These authors further showed that IL-1β induced sST2 secretion from human venous and arterial endothelial cells and suggested that the distinct ability of sST2 to predict HF aside from BNP, which is released by stress, indicates that inflammation may contribute to elevated sST2 levels in HF patients (64). Thus, the consistent finding of elevated sST2 levels in males with various forms of HF suggest that inflammation, and the TLR4-IL-1β pathway in particular, drive the pathogenesis of disease that leads to HF and death.

Limitations of this study include a relatively small sample size, especially for some sub-analyses. For CVDs other than myocarditis obtained from the Mayo Clinic Biobank, we were not able to confirm diagnosis because patients self-reported their disease. However, we selected patients that had echocardiography data available indicating that they had been seen by a cardiologist. Additionally, NYHA class status was available for a significant proportion of the patients further indicating that the Biobank samples were from patients with HF. Another limitation is that we do not know when the initiation of the CVD condition occurred in relation to when the sera was acquired for the Biobank. Future studies should examine whether sex and age differences are found in sST2 levels for these and other CVDs where cases can be confirmed. Age 50 years was used as a cut-off to assess the effect of aging on both men and women, but it remains unclear whether this is the best age cut-off to use to assess the effects of aging in patients with CVD. Another limitation of the study is that we obtained myocarditis samples from various sites mainly in Europe while other CVDs were obtained from the Mayo Clinic Biobank in the US. We also do not have information about medication use or history for the patients and it is possible that certain medications like glucocorticoids could alter sST2 levels. Additionally, myocarditis samples were analyzed using an R&D Systems ELISA kit and converted to Presage kit levels, as previously (4). However, we found that men with myocarditis consistently had significantly higher sST2 levels than women at each study site (data not shown). Additionally, this study provided data on a primarily white population and so may not represent findings in other races and ethnicities.

## 5. Conclusion

In conclusion, in this study of a number of mainly self-reported CVDs, we show that serum sST2 levels are elevated according to sex for myocarditis, cardiomyopathy, and MI, especially in particular age groups. These data indicate that there are important sex and age differences that occur during CVD related to sST2. These findings should be followed up using larger well-defined myocarditis, cardiomyopathy, CAD, MI, and CHF cohorts.

## Data availability statement

The raw data supporting the conclusions of this article will be made available by the authors, without undue reservation.

## Ethics statement

Approval was obtained from the local IRB or ethics board at each institution for studies involving patients. Informed written consent or IRB approved waiver of consent was obtained from all patients.

## Author contributions

KB and DF: conceptualization and project administration. DB, KB, DD, ED, and SS: data acquisition. CT, MC, JK, JB-V, SP, DM, E-SJ, SL, LB, LC, and DF: sample acquisition. DB, KB, and DF: methodology and data curation. DB, KB, DD, and DF: data analysis. DB and DF: writing—original draft. DB, KB, DD, ED, SS, CT, MC, JK, JB-V, SP, DM, E-SJ, SL, LB, LC, and DF: writing—review and editing. All authors have read and agreed to the published version of the manuscript.

## Supplementary material

Supplementary Figure 1 Soluble ST2 (sST2) kit conversion. Twenty one myocarditis samples were run in both the R&D Systems kit (R&D), which was used for all the myocarditis samples and the Critical Diagnostics kit (CD), which was used for all other samples. The conversion factor was calculated to be 2.488 between the two kits. Violin plots denote data distribution for each group, which was unchanged by the conversion.
