# Supplementary figures and images for: Sex and age differences in sST2 in cardiovascular disease
Source: Front Cardiovasc Med. 2023 Jan 18;9:1073814. doi: 10.3389/fcvm.2022.1073814 (PMC9889877; doi:10.3389/fcvm.2022.1073814)

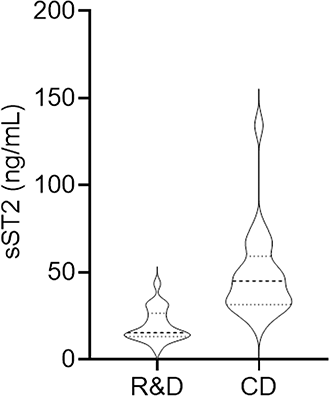

Supplement: Supplementary Figure 1 — Soluble ST2 (sST2) kit conversion. Twenty one myocarditis samples were run in both the R&D Systems kit (R&D), which was used for all the myocarditis samples and the Critical Diagnostics kit (CD), which was used for all other samples. The conversion factor was calculated to be 2.488 between the two kits. Violin plots denote data distribution for each group, which was unchanged by the conversion. [file Image_1.TIF]
